# Supplementary material for: Prediction of Maxillary Bone Invasion in Hard Palate/Upper Alveolus Cancer: A Multi-Center Retrospective Study
Source: Cancers (Basel). 2023 Sep 24;15(19):4699. doi: 10.3390/cancers15194699 (PMC10572084; doi:10.3390/cancers15194699)
Supplement: Supplementary file 1 [file cancers-15-04699-s001.zip › cancers-2621987-supplementary.pdf]

Supplementary Table S1. Comparison of clinical characteristics between the participating centers.

	SMC	AMC	Ajou	*p*-value
Patients No.(%)	84 (58.3)	46 (32.0)	14 (9.7)	144 (100.0)
Age (years, mean ± SD)	57.6 ± 13.3	54.1 ± 16.6	60.0 ±18.2	0.345
Sex (male: female) (No., %)	38: 46 (45.2: 54.8)	23: 23 (50.0: 50.0)	6:8 (42.9: 57.1)	0.837
Histology (No., %) (SCC: minor salivary carcinoma: melanoma)	38: 39: 7 (45.2:46.7:8.3)	13: 26: 7 (28.3:56.5:31.9)	9: 4: 1 (64.3:28.6:7.1)	0.120
Tumor site (No., %) (hard palate: upper alveolus)	67: 17 (79.8: 20.2)	40: 6 (87.0: 13.0)	11: 3 (78.6: 21.4)	0.560
Pathologic bone invasion (No., %)	37 (44.0)	27 (58.7)	6 (42.9)	0.252

Abbreviations: SMC, Samsung Medical Center; AMC, Asan Medical Center; Ajou, Ajou Medical Center; SCC, squamous cell carcinomas.

Supplementary Table S2. MBI prediction models developed by multivariable logistic regression analysis in the patients with primary bone (HP/UA) resection for HP/UA cancer.

	**Predictor variables**	**Model 1 (*n* = 144)**	**Model 5 (*n* = 71)**	**Model 6 (*n* = 44)**
Adjusted odds ratio [95% CI]	CT bone invasion	13.049 [5.829, 29.214]	4.536 [1.076, 19.115]	12.111 [1.238, 118.465]
	Tumor long axis (cut-off value = 2.0, or 1.8 cm)			
	Tumor area (cut-off value = 4.0 cm^2)		3.064 [0.729, 12.875]	4.874 [0.682, 34.823]
	Sex (male vs. female)		2.103 [0.601, 7.361]	2.33 [0.376, 14.425]
	Lymph node metastasis (cN+ vs. cN0)		0.94 [0.268, 3.299]	2.991 [0.378, 23.679]
	PET SUVmax (cut-off value = 6.0)			2.666 [0.356, 19.950]
AUC [95% CI]		0.779 [0.712, 0.847]	0.8045 [0.6879, 0.921]	0.8548 [0.7027, 1.000]
AUC [95% CI] from internal validation			0.7328 [0.4819, 0.973]	0.7118 [0.3453, 0.990]
Brier score [a]		0.168		
P Value by DeLong's test [b]		Ref	0.0310	0.1933

[a] Brier score: the value of the Brier score is between 0.0 and 1.0, where a model with perfect prediction has a score of 0.0 and the worst has a score of 1.0.

[b] Comparison of AUC by DeLong's test

Abbreviations: AUC: Area under receiver operating characteristics curve; CI: confidence interval.

[END]
